# A process-oriented interpretation of perfectionism: error, uncertainty, and the principles of organization

**DOI:** 10.3389/fpsyg.2026.1882582

**Published:** 2026-09-29

**Authors:** Adel Bartos, Kristof Schwartz

**Affiliations:** 1 Doctoral School of Education, ELTE Eötvös Loránd University, Budapest, Hungary; 2 Institute of Health Promotion and Sport Sciences, ELTE Eötvös Loránd University, Budapest, Hungary; 3 Department of Psychology and Health Management, Faculty of Health and Sport Sciences, Széchenyi István University, Győr, Hungary

**Keywords:** conditional self-worth, error sensitivity, perfectionism, phenomenology, uncertainty

## Abstract

Contemporary dimensional models have substantially advanced the psychological understanding of perfectionism. However, growing theoretical and empirical evidence suggests that understanding the phenomenon may also require consideration of additional process-oriented perspectives. Rather than proposing a new definition of perfectionism, the present Perspective introduces a complementary process-oriented interpretative framework that focuses on the principles of organization underlying the phenomenon. Particular attention is directed toward individuals’ relations to error, uncertainty, and performance-related self-evaluation, as well as to the experiential organization associated with these processes. Within this framework, perfectionism is interpreted as a psychological configuration in which flawlessness functions as a normative reference point, organizing self-evaluation, anticipatory regulation, and the interpretation of action. The paper further argues that characteristics commonly associated with perfectionistic strivings may, in some cases, reflect striving for excellence rather than perfectionism itself, suggesting that these forms of functioning may be organized according to different psychological principles. A phenomenological analysis illustrates how anticipation of mistakes may influence attentional focus, temporal experience, self-monitoring, and the lived experience of action. The proposed perspective is not intended to replace existing dimensional models but to complement them by offering an additional process-oriented level of interpretation. At the theoretical level, this framework contributes to a more differentiated understanding of perfectionism, while at the empirical level it highlights new directions for investigating error anticipation, regulatory flexibility, conditional self-evaluation, and other dynamic psychological processes that may not be fully captured by traditional trait-based measures.

## 1 Introduction

Perfectionism is one of the most extensively studied constructs in psychological research, yet it remains conceptually contested (Stoeber, 2018b). Perfectionism has been described as a personality construct involving the pursuit of the best possible performance and a critical evaluation of that performance (Frost et al., 1990). Contemporary literature conceptualizes perfectionism as a multifaceted construct whose facets are frequently subsumed under two broader dimensions of *perfectionistic strivings* and *perfectionistic concerns* (Stoeber and Otto, 2006). Perfectionistic strivings encompass high personal standards and self-oriented perfectionism, whereas perfectionistic concerns are characterized by concerns over mistakes, doubts about actions, socially prescribed perfectionism, perceived discrepancy between one’s expectations and actual performance, and negative reactions to imperfection (Madigan, 2023; Stoeber, 2018b; Stoeber and Otto, 2006). Whereas *perfectionistic concerns* are consistently associated with psychological distress, rigid self-criticism, and fear of failure, the empirical evidence regarding *perfectionistic strivings* is considerably more nuanced (Bieling et al., 2004; Frost et al., 1990; Harper et al., 2016; Hill et al., 2025; Xie et al., 2019). In recent years, however, the interpretation of *perfectionistic strivings* as adaptive has been increasingly criticized because it likely brings together distinct psychological phenomena under a common category (Flett and Hewitt, 2006; Smith et al., 2018). Nevertheless, relatively few theoretical frameworks have approached perfectionism from the perspective of different principles of organization. In this paper, principles of organization do not refer to the conceptual definition or dimensional structure of perfectionism, but to a process-oriented interpretation of the psychological mechanisms associated with its established dimensions, intended to supplement existing trait models.

Perfectionism is approached from the premise that understanding the phenomenon involves more than distinguishing its dimensions or their outcomes (Gaudreau, 2013). Particular attention is therefore paid, in the analysis of the psychological functioning associated with these dimensions, to individuals’ relations to error, uncertainty (Davis and Wosinski, 2012; Howell et al., 2019; Kummer et al., 2024), and performance-related self-evaluation (Ståhlberg et al., 2019). Drawing on findings regarding concern over mistakes, flawlessness may become an implicit norm that shapes both self-evaluation and the interpretation of action (Frost et al., 1997). Accordingly, errors may readily be experienced as deviations from the desired goal state, rather than in terms of their simple, and perhaps neutral, signaling function (Barke et al., 2017), suggesting that examining these processes may contribute to understanding the principles of organization associated with the established dimensions of perfectionism.

The aim of the present paper is to present a complementary interpretative framework that directs attention to the principles of organization and experiential-dynamic aspects of perfectionism. The proposed approach offers analytical perspectives that may contribute to the theoretical interpretation and empirical investigation of the phenomenon.

## 2 Conceptual delimitation

The empirical literature on perfectionism consistently indicates that its different components are associated with qualitatively distinct psychological outcomes (Stoeber et al., 2014; Stricker et al., 2022; Suh et al., 2017). Following the terminology accepted in the literature, the present paper uses the terms *perfectionistic strivings* and *perfectionistic concerns* to refer to the two broader dimensions of perfectionism, whereas *personal standards* and *high standards*, on the one hand, and *concern over mistakes* and *doubts about actions*, on the other, are understood as facets of these respective dimensions. Of these facets, the literature primarily associates those related to the *perfectionistic concerns* dimension with psychopathological symptoms (Bieling et al., 2004; Shafran and Mansell, 2001). The empirical evidence regarding the perfectionistic strivings dimension, however, presents a considerably more nuanced picture (Hill et al., 2018, 2025). Depending on the outcome variables examined, the statistical control of overlap with the perfectionistic concerns dimension, and the situational context of the studies, perfectionistic strivings may show different patterns of association (Hill et al., 2025). According to the literature, under certain conditions, perfectionistic strivings are associated with favorable performance outcomes and lower levels of psychological distress, particularly when it is not accompanied by maladaptive self-evaluative mechanisms (Lizmore et al., 2019; Montano, 2025; Ruiz et al., 2023). In addition, several studies have reported associations with problem-focused coping (Abdollahi et al., 2018; Xu et al., 2024) and approach-oriented motivation (Madigan et al., 2017). Nevertheless, broader meta-analytic evidence also indicates that perfectionistic strivings may show unique positive associations with clinically relevant outcomes (Hill et al., 2025). In a meta-analysis synthesizing 284 studies, perfectionistic strivings showed positive associations with several psychopathological outcomes, including anorexia nervosa and bulimia nervosa, even when its unique contribution relative to perfectionistic concerns was considered (Limburg et al., 2017). Another meta-analysis found a positive association between perfectionistic strivings and orthorexia that remained significant after accounting for their overlap with perfectionistic concerns, whereas the corresponding unique association for perfectionistic concerns was not significant (Pratt et al., 2024). Similarly, perfectionistic strivings were positively associated with the affective component of test anxiety, and this relationship remained significant, although attenuated, after controlling for their overlap with perfectionistic concerns (Burcaş and Creţu, 2021). Consistent with this, Smith and colleagues (Smith et al., 2018), based on a meta-analytic review of 45 studies, concluded that the association between perfectionistic strivings and suicidal ideation calls into question the assumption that such strivings can, in themselves, be regarded as healthy. These observations raise the question of whether perfectionistic strivings and perfectionistic concerns are characterized by different psychological processes, whether these processes also help distinguish striving for perfection from striving for excellence, and whether such a process-oriented framework may meaningfully complement current dimensional approaches to trait perfectionism. Characteristics commonly associated with perfectionistic strivings - such as high standards - should not necessarily be interpreted as more favorable forms of perfectionism. Rather, they may, in some cases, reflect striving for excellence or achievement-oriented work style rather than perfectionism itself (Flett and Hewitt, 2002; Greenspon, 2008). Accordingly, the difference between perfectionism and striving for excellence is structural in nature. This perspective is also consistent with contemporary models that conceptualize striving for perfection and striving for excellence as conceptually distinct constructs and assume differences in both their functional characteristics and developmental patterns (Gaudreau et al., 2022). Whereas perfectionistic concerns are associated with control-oriented responding (Gerdan and Salcioglu, 2025) and conditional self-evaluation (Ståhlberg et al., 2019), striving for excellence permits flexible goal pursuit without requiring flawlessness (Gaudreau, 2019; Gaudreau et al., 2023). Taken together, these distinctions raise the question of whether perfectionistic strivings and perfectionistic concerns themselves should be interpreted as reflecting the same organizational principles. It is well established that these two phenomena do not demonstrate consistent empirical co-occurrence and may be associated with different psychological outcomes and mediational mechanisms (Gotwals et al., 2012). If one phenomenon is associated with psychopathological symptoms, distress, and rigid control tendencies, whereas the other is associated with flexibility, improved performance, and lower levels of distress, this raises the question of whether these phenomena may be more appropriately understood from different organizational perspectives.

## 3 Perfectionism as an ontological configuration

Perfectionism has been conceptualized within several theoretical frameworks. Personality psychology generally conceptualizes perfectionism as a multidimensional construct (Gaudreau, 2013; Stoeber, 2018a; Taylor et al., 2016), whereas clinical models approach it as a transdiagnostic process, namely a maladaptive cognitive-behavioral pattern that may contribute to the development and maintenance of multiple forms of psychopathology (Gerdan and Salcioglu, 2025). The present paper does not seek to redefine the ontological classification of perfectionism, but instead directs attention to the internal organization of the phenomenon. In this context, the term “ontological” refers to the interpretative mode through which individuals relate to error, uncertainty, and imperfection rather than to its metaphysical meaning.

The psychological processes considered in this section are primarily informed by clinical models of perfectionism and the literature on perfectionistic concerns. This perspective is consistent with approaches that associate perfectionism with conditional self-worth (Hill et al., 2011; Ståhlberg et al., 2019), maladaptive schemas (for example, beliefs such as “I am fundamentally flawed,” “I am inadequate,” or “I must always be perfect”), and metacognitive control processes (Shafran et al., 2002). The present paper, however, places emphasis on the way these psychological characteristics are organized through individuals’ mode of relating to error, uncertainty, and imperfection, rather than on their mere presence. The same behavioral manifestations - such as intensive practice or persistent work engagement - may carry different meanings depending on the underlying mode of relating associated with them: in one case, they may reflect development or striving for excellence, whereas in another they may become instruments for avoiding mistakes and maintaining self-worth.

From this perspective, perfectionism may be described as a configuration in which the inherent imperfection of human functioning appears as a characteristic requiring correction. Accordingly, flawlessness becomes the normative reference point through which experience is organized, such that errors are experienced as deviations from a desired goal state rather than as forms of learning-related feedback (Barke et al., 2017). This may further contribute to an increased need for control, rigidity within regulatory processes, and a progressively tighter association between self-evaluation and performance outcomes (Hill et al., 2011; Shafran et al., 2002; Shafran and Mansell, 2001). In this sense, perfectionism may be understood as a psychological configuration emerging from a particular attribution of meaning to human imperfection.

## 4 The phenomenological structure of perfectionism

Within the perspective proposed in the present paper, the phenomenological processes described below are expected to characterize perfectionistic concerns, and potentially clinically relevant forms of perfectionistic strivings, rather than healthy striving for excellence. Given the heterogeneous and context-dependent associations reported for perfectionistic strivings (Hill et al., 2025), it remains an open empirical question whether these experiential processes emerge similarly across the two dimensions. Accordingly, the following analysis should be understood as a process-oriented interpretative framework from which empirically testable hypotheses may be derived regarding how these processes differ across perfectionistic concerns, perfectionistic strivings, and striving for excellence.

From a phenomenological perspective, perfectionistic concerns may also be grasped through the structure of subjective experience itself. Within this framework, attentional focus, the experience of temporality, and self-monitoring during action may display a pattern in which the anticipation of possible mistakes assumes a central role. Under such conditions, the possibility of error pre-structures present action, which may consequently appear as a potential source of failure (Frost et al., 1990; Sagar and Stoeber, 2009). Accordingly, the future is constituted less as a field of open possibilities and more as a horizon defined by the necessity of correction. This dynamic may be related to classical phenomenological analyses of time-consciousness, according to which the present is not understood as an isolated moment in time, but rather as the dynamic unity of anticipation (protention) and retention (Husserl, 2000; Komorjai, 2017). Within the context of perfectionistic concerns and potentially clinically relevant forms of perfectionistic strivings, this protentional dimension may become increasingly oriented toward the anticipation of possible mistakes, thereby altering the lived experience of present action itself: for example, during the execution of a task, the need to avoid mistakes may become especially salient, potentially at the expense of direct involvement in the activity. Under such conditions, the perceptual and action-related framework of behavior may also be altered. Whereas action may ideally unfold as a form of practical openness, flexibly adapting to the demands of the task, within a context of perfectionistic concerns this openness may become constricted (Goulet-Pelletier et al., 2022), particularly when heightened sensitivity to possible mistakes comes to the foreground (Shafran et al., 2002). As a consequence, action may become less a spontaneously unfolding process and more an activity subjected to continuous monitoring and control (Horney, 1950; Saboonchi and Lundh, 1999). This constriction may emerge not only at attentional or cognitive levels, but also as a bodily experience. It may manifest, for example, in the more controlled execution of movements, disruption of the natural rhythm of action, or increased muscular tension (Ioannou et al., 2016).

From the standpoint of phenomenological description, the retentional dimension is likewise significant. When previous mistakes or experiences of inadequacy persist not merely as completed events, but as experiential traces that continue to shape present self-interpretation, they may contribute to the organization of action around heightened self-monitoring and anticipatory sensitivity from the outset. In this sense, the temporal structure of experience associated with perfectionistic concerns may be constituted not only through anticipation of future mistakes, but also through retained experiences of the past, particularly insofar as these are connected to the vulnerability of self-evaluation (Hill et al., 2011; Ståhlberg et al., 2019). Such experience may therefore be identified as more than an intensified performance focus; it may be understood as a temporally structured mode of experience in which retained traces of the past and anticipation directed toward the future jointly shape the lived experience of present action.

## 5 Theoretical and empirical implications

The present perspective may have theoretical and empirical implications at multiple levels. At the theoretical level, it draws attention to the possibility that, alongside the dimensional interpretation of perfectionism, consideration of additional organizational perspectives may also be warranted (Gaudreau, 2013), which may contribute to a more differentiated theoretical interpretation of the phenomenon. Classical models of perfectionism - including multidimensional and component-based approaches - have identified important distinctions among different aspects of the construct; nevertheless, these aspects continue to be treated as elements within the same conceptual framework (Bieling et al., 2004; Frost et al., 1990; Hewitt and Flett, 1991; Smith et al., 2022; Stoeber and Otto, 2006). The present approach, however, raises the possibility that the interpretation of certain phenomena subsumed under perfectionism may also warrant consideration of additional organizational perspectives. Accordingly, the theoretical interpretation of dimensional models may require further refinement in certain respects, particularly with regard to the interpretation of the organizational characteristics of the different phenomena associated with perfectionism.

At the empirical level, these considerations may also justify a reconsideration of existing measurement approaches to perfectionism. Widely used scales - such as the various forms of the Multidimensional Perfectionism Scale (Hewitt and Flett, 1991), the Frost Multidimensional Perfectionism Scale (Frost et al., 1990), or the Almost Perfect Scale-Revised (Slaney et al., 2001) - treat heterogeneous components within a shared measurement framework, which may contribute to inconsistent interpretations of empirical findings. One consequence of this is that the dimension of perfectionistic strivings has been associated with positive outcomes in some studies (Stoeber et al., 2007, 2008; Stoeber and Rambow, 2007), whereas certain dimensions of perfectionism have been associated with a range of maladaptive outcomes (Anshel and Mansouri, 2005; Curran and Hill, 2019; Smith et al., 2018; Xie et al., 2019). Such discrepancies may therefore reflect not merely empirical inconsistency, but also the heterogeneous conceptualization of the construct itself. In addition, dominant measurement approaches are typically based on self-report, trait-level operationalizations, which possess limited capacity to capture the temporal and processual characteristics of the different forms of perfectionistic functioning.

Consequently, it may be particularly relevant to investigate process-oriented dimensions in a more differentiated manner, including the degree of error anticipation, the intensity of self-monitoring during action, the conditionality of self-evaluation, and regulatory flexibility. Of particular relevance may be the extent of error anticipation, which may be associated with future-oriented anxiety; the conditionality of self-evaluation, which may mediate the relationship between perfectionistic concerns and psychological distress (Xie et al., 2019); and regulatory flexibility, which may allow for the examination of the context-dependent organization of these patterns. These phenomena may not be captured with sufficient precision through traditional questionnaire-based trait assessments, thereby justifying the application of methodological approaches more sensitive to temporal and contextual dynamics, such as experimental paradigms, experience sampling methodologies, or longitudinal designs. This perspective suggests that a more precise empirical understanding of perfectionism may require not only the identification of static characteristics, but also the more direct examination of dynamic psychological processes.

## 6 Discussion

The present paper argued that the dimensional interpretation of perfectionism alone does not fully capture the conceptual complexity of the phenomenon. Based on the present analysis, it may be suggested that the interpretation of certain phenomena currently understood within the common conceptual framework of perfectionism may also warrant consideration of additional organizational perspectives. A more nuanced understanding of perfectionism may therefore require not only the differentiation of the construct’s components, but also an approach that is more sensitive to its context-dependent and temporal characteristics. The present perspective does not seek to invalidate existing models of perfectionism; rather, it offers a complementary interpretative framework that directs attention toward the principles of organization and experiential-dynamic aspects of the phenomenon.

## Data Availability

The original contributions presented in the study are included in the article/supplementary material, further inquiries can be directed to the corresponding author.
